# Breast osteosarcoma originating from a malignant phyllodes tumor: A case report of a rare neoplasm

**DOI:** 10.1097/MD.0000000000027908

**Published:** 2021-11-24

**Authors:** Ying Jin, Lirong Bi, Ruming Yang, Tinghan Jiang, Xiaoxiao Zhang, Sijie Li

**Affiliations:** a Department of Breast Surgery, First Hospital of Jilin University, Changchun, Jilin, People's Republic of China; b Department of Pathology, First Hospital of Jilin University, Changchun, Jilin, People's Republic of China.

**Keywords:** case report, malignant phyllodes tumor, osteosarcoma

## Abstract

**Rationale:**

Malignant phyllodes tumors with osteosarcomatous transformation are exceedingly rare. The clinical manifestations are similar to those associated with benign giant calcification, resulting in nonspecific and complex clinical symptoms.

**Patient concerns:**

A 59-year-old woman presented with a firm, painless, movable, 5.0 × 4.0 cm lump in the lower inner quadrant of the left breast that she had detected 1 month prior.

**Diagnoses:**

Breast osteosarcoma originating from a malignant phyllodes tumor was confirmed by histopathologic and immunohistochemical evaluation.

**Interventions:**

The patient underwent a wide local excision.

**Outcomes:**

The patient recovered uneventfully and was discharged after the operation. The 6-month postoperative follow-up assessment revealed no evidence of recurrence.

**Lessons:**

Diagnosing malignant phyllodes tumors with osteosarcomatous transformation requires a high level of suspicion and awareness by both surgeons and pathologists. They should be aware of the extent of such disease, which might be mistaken as benign giant calcification. Medical history and imaging findings are important for accurate diagnosis. Phyllodes tumor with an osteosarcomatous component is an aggressive neoplasm associated with distant metastasis. Delayed diagnosis and insufficient excision might negatively impact both treatment and survival.

## 1 Introduction

Phyllodes tumors (PTs), which account for less than 1% of mammary gland tumors, are rare fibroepithelial lesions of the breast. PTs have both epithelial and stromal components and can be classified as benign, borderline, or malignant.^[1]^ Malignant phyllodes tumors (MPTs) account for 10% to 30% of PTs. Despite being benign, PTs might acquire malignant features. At presentation, 80% of MPTs are localized; 8.2% are associated with regional disease, lymph node involvement, or direct extension into adjacent tissue; and 1.5% are associated with metastatic disease.^[2]^ Clinical presentation is often preceded by rapid tumor growth, but growth rate has not been firmly associated with malignancy. Tumor diameters vary between 4 and 7 cm. Ulceration and fixation to the chest wall are rare even when these tumors are malignant.^[3]^

MPTs are characterized by marked stromal cellularity, nuclear atypia, stromal growth, more than 10 mitoses per high-power field, and infiltrative tumor margins. Moreover, the presence of heterologous sarcomatous elements within the tumor, such as liposarcoma, chondrosarcoma, or osteosarcoma, directly qualifies PTs as malignant regardless of other histopathologic features.^[4]^ MPTs with osteosarcomatous transformation are exceedingly rare and are often initially misdiagnosed as benign giant calcifications. Different imaging modalities and pathologic features play important roles in differentiating breast osteosarcoma from other benign and malignant lesions of the breast with drastically different management strategies. To add to the clinical evidence regarding this rare entity, we describe a woman diagnosed with breast osteosarcoma (originating from an MPT) based on detailed imaging and histopathologic records, and we review the literature to describe typical imaging features to aid diagnosis.

## 2 Case report

**Figure 1:**
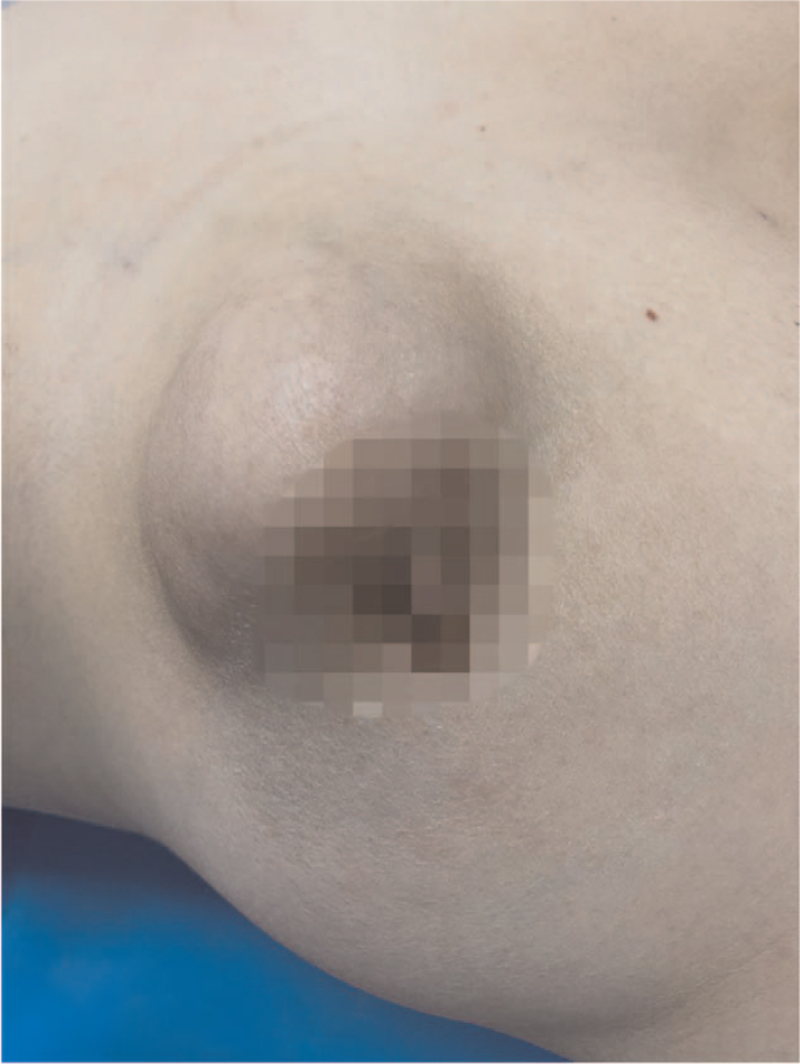
Physical examination. The hard mass on the left breast, which is 5.0 × 4.0 cm in size. The boundary is unclear, the shape is irregular, and the mass is movable.

**Figure 2:**
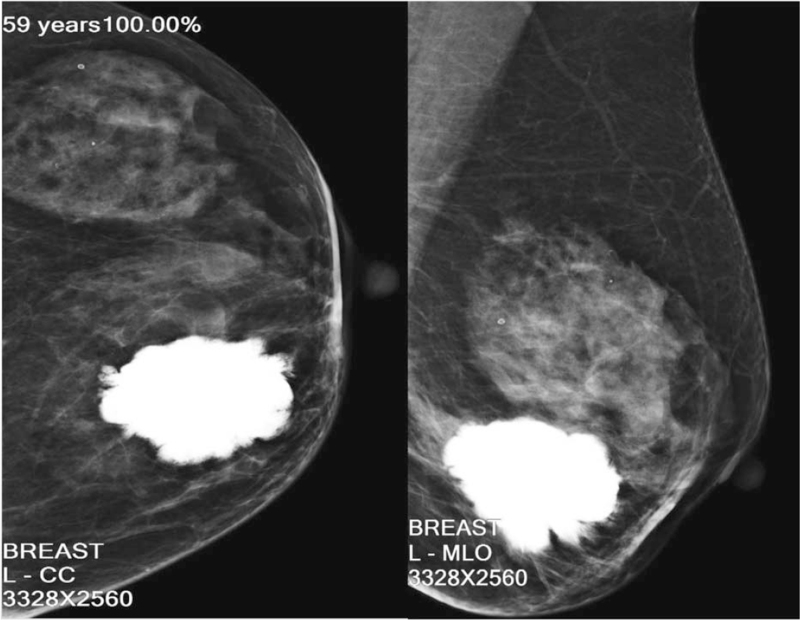
Left mammogram: in the lower internal quadrant, a solid nodule with partially defined lobulated edges is observed.

**Figure 3:**
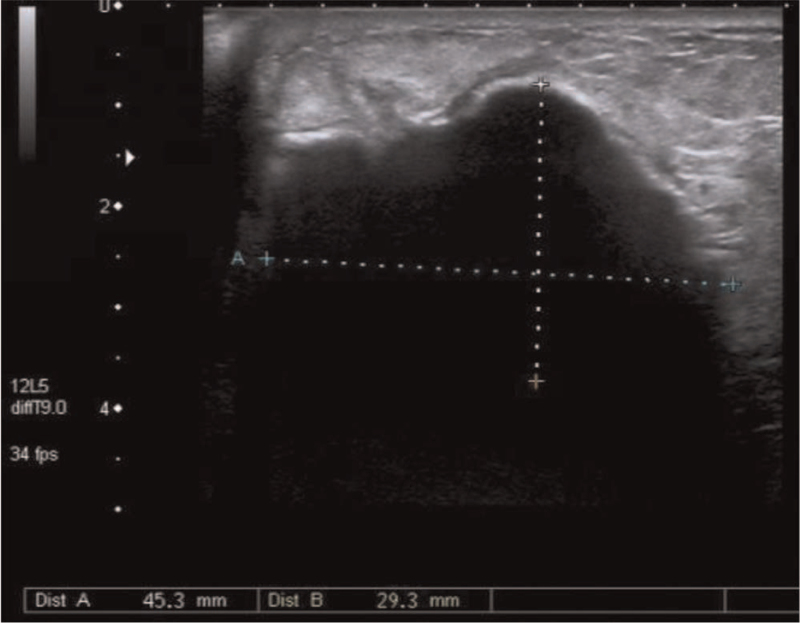
Ultrasonography. A 45.3 mm-diameter relatively smoothly marginated hypoechoic mass containing multiple calcifications.

**Figure 4:**
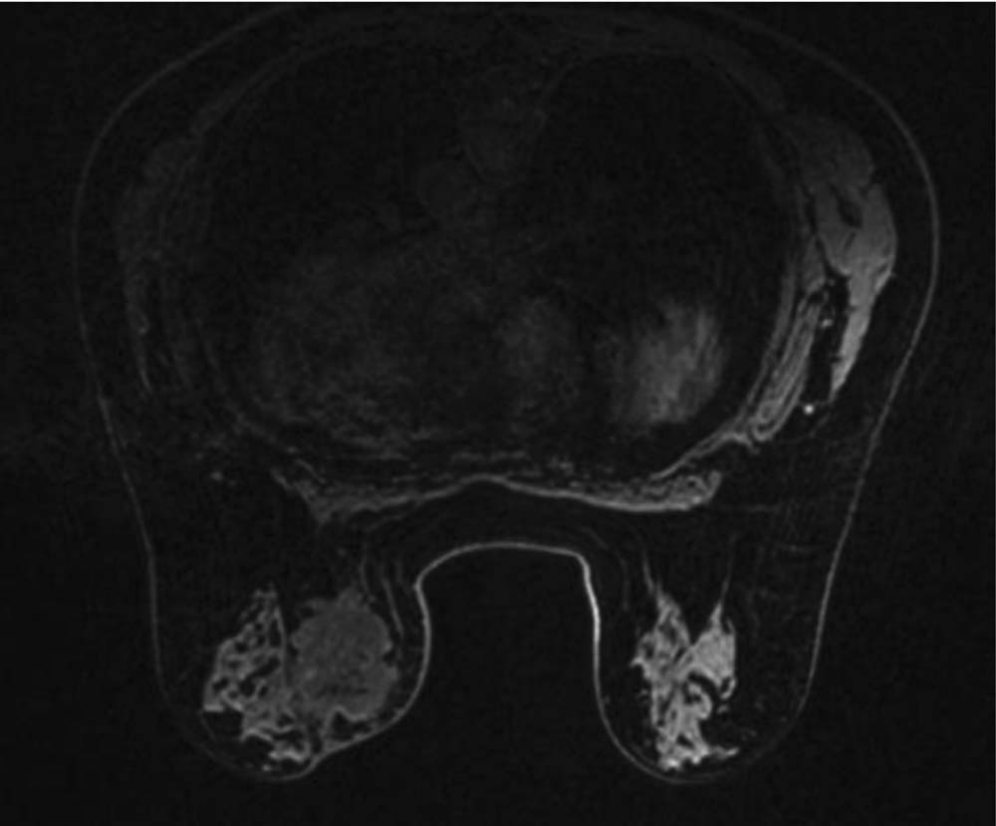
MRI. A 3.7 × 5.6 × 4.7 cm mass at the lower inner portion of the left breast. MRI = magnetic resonance imaging.

A 59-year-old woman with no family history of breast cancer presented with a breast lump that she detected 1 month previously. She denied hormonal therapy intake, she was postmenopausal, and she experienced menarche at 12 years of age. She described initially palpating a nodule in her left breast 1 month before presenting and noticing that the lesion increased in size over the next 3 weeks. The patient then visited our hospital for further treatment. Physical examination revealed a 5.0 × 4.0 cm, firm, irregular, palpable lump in the lower internal quadrant of the left breast. The mass was not attached to the muscles and it was not associated with any skin changes. There were no palpable axillary lymph nodes (Fig. 1). The contralateral breast and armpit were unremarkable with no palpable masses or lymph nodes. Mammography revealed a hyperdense nodule with irregular borders, which was associated with slight retraction of the adjacent tissue (Breast Imaging Reporting and Data System [BI-RADS] 3) (Fig. 2). Ultrasonography revealed a solid nodule, and there was no internal vascularity upon application of color Doppler scanning; the nodule measured 45.3 × 29.3 mm at its largest diameter (BI-RADS 4) (Fig. 3). Contrast-enhanced magnetic resonance imaging (MRI) revealed a 3.7 × 5.6 × 4.7 cm mass. T1-weighted imaging yielded equal or slightly higher signals, T2-weighted imaging yielded uneven or slightly higher signals, and diffusion-weighted imaging yielded edge hyperintensity. An enhanced scan showed uneven enhancement, and the edge of the lesion was irregular and lobulated, with visible burrs. The lesion spread to the front of the pectoralis major muscle. The local pectoralis major muscle T2, T1, and fat pressure images showed a flaky high signal, and the time–signal curve was of the rapidly increasing–decreasing type (BI-RADS 5) (Fig. 4). Chest and abdominal computed tomography examinations did not reveal any signs of distant tumor metastasis.

**Figure 5:**
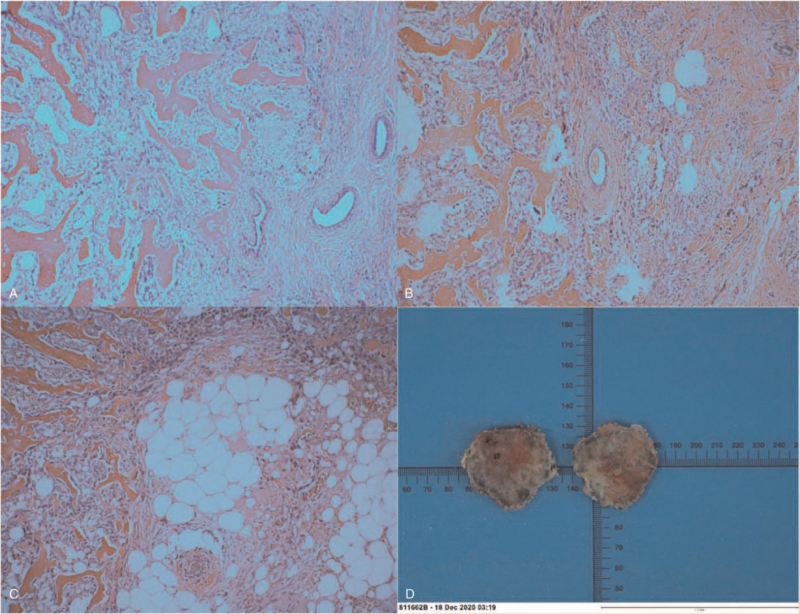
The histopathological examination. (A) Low grade osteosarcoma invading the breast tissue. (B) Residual mammary duct can be seen in osteosarcoma. (C) Osteosarcoma invading the breast adipose tissue. (Hematoxylin-eosin, 100×) (D) Specimen.

The patient underwent wide local excision, and the mass indeed did not involve the thoracic muscles. Paraffin section examination confirmed a diagnosis of osteosarcoma with an osteoblast component originating from an MPT (tumor size 5.5 × 4.0 × 3.5 cm) (Fig. 5D). The tumor was mainly composed of osteosarcoma, with well-differentiated trabeculae in the central area, which were connected to each other. There were no stretching or expanding breast ducts, and a slightly dilated benign duct could be seen. Osteosarcoma cells at the edge of the tumor showed obvious atypia, increased cell density, accompanied by pushing and infiltrating growth mode (Fig. 5A-C). CKpan expression was negative both in sarcomatous areas and in benign ductal epithelium. SATB2 expression was diffusely positive in the osteosarcoma cell nuclei. The cell proliferation index Ki67 of the sarcomatous area was high (40%), with accompanying expression of the myogenic marker SMA in the sarcomatous area.

The patient refused any additional chemotherapy or external radiotherapy after surgery, and close follow-up was prescribed. We conducted a half-year follow-up, and the patient showed no signs of relapse.

## 3 Discussion

PTs account for less than 1% of mammary gland tumors, have both epithelial and stromal components, and can be classified as benign, borderline, or malignant.^[1]^ On ultrasound, PT features include the presence of fluid-filled spaces, lobulations, and marked posterior acoustic enhancements.^[5]^ On mammography, a PT typically appears as a well-circumscribed, hyperdense or isodense, round, or ovoid mass.^[6]^ On MRI, PTs appear ovoid, well-circumscribed, isointense on T1-weighted images, and heterogeneously hyperintense on T2-weighted images. MRI characteristics that favor malignant over benign PTs include the following: cystic changes with an irregular wall and tumor signal intensity lower than or equal to normal fibroglandular tissue on T2-weighted images.^[7]^ In this case, the lesion was initially graded as a benign giant calcification based on mammography and ultrasonography images. However, we finally performed a wide local excision due to the rapid growth of the lesion and the BI-RADS 5 MRI result. We retrospectively examined the characteristics of this case after the histopathological examination. Usually, “coarse or popcorn-like” or “round” calcifications indicate benign calcifications.^[8]^ Our patient's mammogram showed an irregular and coarse mass with a spiculated margin; this morphology should raise the suspicion of the presence of malignancy. Histopathologically, MPTs are diagnosed when all of the following features are present: marked stromal nuclear pleomorphism, stroma overgrowth, increased mitosis, increased stromal cellularity, and an infiltrative border.^[9]^ This neoplasm must be differentiated from metaplastic carcinoma. In our case, the immunohistochemical results—CKpan (-) and p63 (-)—did not support a diagnosis of metaplastic carcinoma.

Breast osteosarcoma diagnosis and treatment remain controversial owing to a lack of high-level data and the absence of prospective studies. Based on the available evidence, the standard treatment is mainly surgical, with complete excision of the tumor with wide margins or total mastectomy. Axillary tumor involvement occurs in less than 5% of patients; therefore, axillary surgical exploration is not routinely recommended unless imaging report showed abnormal lymph node. The roles of radiotherapy and chemotherapy remain unclear.^[10]^ PT recurrences are rare after excision of benign PTs, but recurrence of borderline and malignant PTs is common; such recurrences are usually due to a positive resection margin status. Large tumor size, stromal overgrowth, high tumor count, nuclear atypia, and pleomorphism are considered risk factors for local recurrence.^[11]^ Silver et al^[12]^ found that PTs with osteosarcomatous components were potentially aggressive neoplasms with distant metastases (lung, brain, contralateral breast) and tumor-related death occurring in 38% and 33% of patients, respectively. Both tumor size and the histologic osteosarcoma subtype significantly correlated with prognosis, with decreased disease-specific survival associated with tumors >5 cm and those exhibiting an osteoclastic or osteoblastic subtype; the investigators concluded that the osteosarcomatous component increased mortality by 33%. Kapiris et al^[13]^ found that MPTs exhibit higher local recurrence (12%-65%) and metastatic (up to 27%) rates, with larger tumors and inadequate surgical margins. Replacement of the stromal component with osteosarcomatous or chondrosarcomatous histological features makes the prognosis even worse. Some evidence suggests that MPTs with heterologous osteosarcomatous differentiation are more aggressive than typical tumors of this type, but compared with osteosarcomas in general, they have a much lower risk of metastasis.^[14]^

## 4 Conclusion

Our case report illustrates that breast osteosarcoma originating from an MPT is remarkably difficult to diagnose and manage. To facilitate early diagnosis, surgeons must combine clinical findings, imaging techniques (ultrasound, computed tomography, MRI), and medical history. The standard treatment comprises complete excision of the tumor with wide margins or total mastectomy.

## Author contributions

**Funding acquisition:** Sijie Li.

**Investigation:** Ruming Yang, Tinghan Jiang, Xiaoxiao Zhang.

**Supervision:** Sijie Li.

**Visualization:** Lirong Bi.

**Writing – original draft:** Ying Jin.

**Writing – review & editing:** Sijie Li.
